# Pharmacokinetics of Danofloxacin in Gushi Chickens after Single Oral and Intravenous Administration

**DOI:** 10.3390/metabo13080906

**Published:** 2023-08-02

**Authors:** Jun-Cheng Chen, Fang Yang, Guang-Hui Li, Ming-Hui Duan, Ze-En Li, Yan Dai, Mei Zhang, Fan Yang

**Affiliations:** 1 College of Animal Science and Technology, Henan University of Science and Technology, Luoyang 471023, China; chenjuncheng@stu.haust.edu.cn (J.-C.C.);; 2 Shantou Customs District Technology Center, Shantou 515031, China

**Keywords:** danofloxacin, domestic chickens, Gushi chickens, pharmacokinetics, HPLC

## Abstract

This study aimed to determine the pharmacokinetics of danofloxacin in Gushi chickens after a single oral (PO) and intravenous (IV) dose at 5 mg/kg body weight (BW). Thirty-two Gushi chickens, aged 20 weeks, were selected and divided into two groups at random, with each group consisting of 16 chickens, evenly distributed between males and females. Following danofloxacin administration, blood samples were taken at predetermined time intervals and the plasma was separated. The concentrations of danofloxacin in plasma were quantified by HPLC with a fluorescence detector. Then the concentrations versus time data were subjected to non-compartmental analysis (NCA) using Phoenix software (version: 8.1.0). After administering danofloxacin orally at a dose of 5 mg/kg BW to Gushi chickens, our results demonstrated that the peak concentration reached 0.53 μg/mL at 4 h. The half-life of absorption (t_1/2ka_) was determined to be 2.37 ± 1.60 h, and the bioavailability (F) was calculated as 40.12 ± 15.83%. For both oral and intravenous administration, the area under the concentration–time curve (AUC_0-∞_) was determined to be 4.72 ± 1.86 and 11.76 ± 3.25 h·µg/mL, respectively. The corresponding elimination half-life (t_1/2λz_) was measured as 11.24 ± 3.90 and 10.17 ± 3.72 h. Moreover, the mean residence time (MRT) was calculated as 10.20 ± 2.47 and 7.05 ± 1.97 h for these respective routes. Based on the calculated AUC/MIC ratio values, it can be inferred that the 5 mg/kg BW dosage of danofloxacin, whether administered orally or intravenously, is expected to effectively treat *Escherichia coli* and *Pasteurella multocida* infections in Gushi chickens.

## 1. Introduction

Gushi chickens, a popular breed in China known for their high-quality meat and eggs, play a crucial role in poultry production [1]. Due to their distinct flavor and nutritional value, Gushi chickens have gained significant consumer appeal. Additionally, they are frequently utilized as breeding hybrids [2]. As the demand for Gushi chickens surpasses their availability, there is a pressing need to address the challenges associated with their production process. Unlike cage breeding, Gushi chickens are often raised in a free-range environment, which increases the risk of infectious diseases. This heightened susceptibility makes them particularly vulnerable to large-scale outbreaks of colibacillosis. These infections not only jeopardize chicken quality but also pose a direct threat to economic profits, potentially leading to substantial loss of the entire flock. Consequently, it is imperative to explore effective antimicrobial drugs for the treatment of Gushi chickens. Undoubtedly, addressing the issue of antimicrobial treatment in Gushi chickens holds significant importance, considering both the economic implications and the preservation of chicken health.

Fluoroquinolones are a rapidly developing class of synthetic antibacterial compounds [3]. They work by inhibiting bacterial DNA gyrase and topoisomerase IV, preventing the normal replication of bacterial DNA and leading to irreversible damage, thus exhibiting antibacterial effects [4]. These compounds are widely used in the treatment of various bacterial infectious diseases in livestock and poultry due to their advantages such as long half-life, low plasma protein binding rate, strong bactericidal activity, ease of clinical use, and minimal side effects [5].

Danofloxacin, a third-generation veterinary fluoroquinolone, is frequently used in veterinary clinics in its mesylate salt form. It possesses broad-spectrum antibacterial activity against most Gram-negative bacteria, some Gram-positive bacteria, as well as certain mycoplasma and chlamydia species [6]. Danofloxacin exhibits excellent lung tissue permeability and high sensitivity to lung infection bacteria. Compared to earlier quinolones, it offers the advantages of high efficiency and low toxicity [7], making it a common treatment choice for various diseases such as colibacillosis, mycoplasma infection, and pneumonia, among others, in diverse animal species. Additionally, danofloxacin is often employed in control programs owing to its remarkable therapeutic effects [8].

Numerous studies have examined the pharmacokinetics of danofloxacin in various bird species, including California brown pelicans, common pheasants, guinea fowls, Japanese quails, and Bilgorajska geese [9,10,11]. However, there is a lack of research on the pharmacokinetics of danofloxacin specifically in Chinese domestic chicken breeds, especially in Gushi chickens. Studying the pharmacokinetics of danofloxacin in Gushi chickens is of great long-term significance. It can help prevent and treat related diseases, as well as contribute to the breeding and development of local chicken breeds. Therefore, the objective of this study is to explore the oral and intravenous pharmacokinetics of danofloxacin in Gushi chickens at a dose of 5 mg/kg BW.

## 2. Materials and Methods

### 2.1. Drugs and Reagents

The danofloxacin mesylate reference substance (Lot No. h0201210; purity of 94.2%) was obtained from the China Institute of Veterinary Drugs Control (Beijing, China), while the danofloxacin mesylate raw material (Lot No. 201217-1; purity of 95.37%) was sourced from Guobang Pharmaceutical Co., Ltd. (Hangzhou, China). Phosphoric acid (H_3_PO_4_) was purchased from Shanghai Boer Chemical Reagent Co., Ltd. (Shanghai, China), and tri-ethylamine was acquired from Shanghai Yien Chemical Technology Co., Ltd. (Shanghai, China). Methanol (chromatographic grade) and acetonitrile (chromatographic grade) were obtained from Shanghai Macklin Biochemical Technology Co., Ltd. (Shanghai, China). Additionally, a 0.9% sodium chloride injection was purchased from Anhui Fengyuan Pharmaceutical Co., Ltd. (Hefei, China). All other reagents were obtained from commercial sources.

### 2.2. Animals

We obtained a total of 32 healthy Gushi chickens (half male, half female) at the age of 20 weeks from a commercial farm in Luoyang City. Each chicken was individually placed in a wire cage measuring 55 × 55 × 45 cm, which was equipped with an automatic water dispenser. The Gushi chickens were provided with an antimicrobial-free diet and had unrestricted access to water. The feeding environment was maintained at a temperature of 21 ± 0.6 °C, with proper ventilation, and a daily light exposure of 16 h. Before any procedures were conducted, the chickens were given a two-week acclimation period. The study protocol received approval from the Institutional Animal Care and Use Committee (IACUC) at Henan University of Science and Technology, with the approval number DK20230303.

### 2.3. Experimental Design and Sample Collection

In our study, we initially weighed 32 Gushi chickens and then randomly divided them into two groups: oral (PO) and intravenous (IV). These groups consisted of equal numbers of male and female chickens. The body weights of the PO and IV groups ranged from 1.36 to 2.53 kg and 1.33 to 2.57 kg, respectively. To administer the oral dose, we prepared a 5 mg/mL solution of danofloxacin mesylate raw material using purified water. The chickens in the PO group were given a dose of 5 mg/kg BW through a gavage tube. Venous blood samples were collected from the wing veins at specific time intervals: 10, 15, 30 min, 1, 1.5, 2, 4, 6, 8, 12, 24, 36, and 48 h after administration. For the intravenous treatment, we prepared an injection solution of 20 mg/mL danofloxacin mesylate raw material using 0.9% sodium chloride. The IV group received a dose of 5 mg/kg BW, which was administered intravenously through the left wing vein. Venous blood samples were collected from the right wing vein at specific time intervals: 5, 15, 30, and 45 min, and 1, 1.5, 2, 4, 6, 8, 12, 24, 36, and 48 h after administration. Approximately 1 mL of blood was collected at each time point and then processed by centrifugation at 4000× *g* for 10 min. The resulting plasma was transferred to 1.5 mL centrifuge tubes. All plasma samples were stored at −20 °C until further analysis.

### 2.4. Determination of Drug Concentrations

Danofloxacin concentrations were determined using a previous HPLC method [12]. Briefly, 0.5 mL of plasma was thoroughly mixed with 1 mL of acetonitrile for 3 min using a vortex. Subsequently, the mixture was centrifuged at 12,000× *g* for 10 min. The supernatant was carefully collected in a clean glass tube and the extraction process was repeated. All extract was then dried using a nitrogen gas flow at 50 °C. The residue was dissolved in 2 mL of the mobile phase and vortexed for 1 min. The liquid was filtered through a 0.22-μm filter into an autosampler glass vial. Finally, 20 μL of the supernatant was analyzed using the HPLC system.

The concentration of danofloxacin was determined using the Waters e2695 HPLC system, which was connected to a 2475 fluorescence detector and controlled by Empower software (version: 3471. Waters Corporation, Milford, MA, USA). The separation process utilized a Hypersil BDS C18 column (4.6 mm × 250 mm, 5 μm, Elite Analytical Instruments Co., Ltd.; Dalian, China). Each sample (20 μL) was injected, and the column was maintained at 30 °C for elution over a duration of 12 min. Fluorescence detection was carried out with an excitation wavelength of 280 nm and an emission wavelength of 450 nm. The mobile phase, consisting of a 0.05 mol/L phosphate buffer (adjusted to a pH of 2.6 with triethylamine) and acetonitrile (*v*:*v* = 83:17), was delivered at a flow rate of 1 mL/min.

A standard stock solution of danofloxacin (500 μg/mL) was prepared using methanol and stored in the dark at 4 °C. This solution served as the basis for standardization and verification of the analytical method. The standard working solution was freshly prepared by diluting the standard stock solution with the mobile phase and used immediately. The standard curve was created through linear regression analysis by mixing a 450 μL aliquot of drug-free plasma obtained from healthy Gushi chickens with 50 μL of a standard working solution containing various concentrations of danofloxacin. The concentrations used for the standard curve were 0.005, 0.01, 0.05, 0.1, 0.5, 1, 2, and 5 μg/mL. The linear correlation coefficient (R^2^) was further determined for the standard curve. To assess the coefficient of variation and recovery, triplicate samples containing low, medium, and high concentrations of danofloxacin (0.01, 0.5, and 2 μg/mL) were spiked to plasma. This process was repeated three times a day for a total of three days. The limit of detection (LOD) and limit of quantification (LOQ) were determined based on signal-to-noise (S/N) ratios of ≥3 and ≥10, respectively.

### 2.5. Pharmacokinetic Analysis

The concentration–time data of danofloxacin in each Gushi chicken plasma were analyzed using the non-compartment model analysis (NCA) method in Phoenix WinNonLin software (version 8.1; Pharsight, Cary, NC, USA) to obtain pharmacokinetic parameters. For both routes of administration, the first-order rate constant associated with the terminal phase (λz) was calculated using linear regression, from which the terminal half-life (t_1/2λz_) was determined as ln2/λz [13]. The area under the concentration–time curve (AUC) and the first moment curve (AUMC) were calculated using the linear trapezoidal rule with extrapolation to infinity [14]. AUC% represents the ratio of AUC_last-∞_ to AUC_0-∞_. The mean residence time (MRT) was calculated as MRT = AUMC_0-∞_/AUC_0-∞_. For the IV route, the initial concentration (C_0_) was estimated using the back extrapolation method. Total body clearance (Cl) was determined as the ratio of the intravenous dose to AUC [15], while the volume of distribution (V_Z_) was calculated as V_Z_ = Dose/AUC/λz, where Dose represents the intravenous dose. The volume of distribution at steady state (V_SS_) was determined as V_SS_ = MRT_IV_ × Cl. For PO administration, the peak concentration of danofloxacin (C_max_) and the time to reach it (T_max_) were both directly read from the concentration versus time curve [16]. The mean absorption time (MAT) was calculated as MRT_PO_ minus MRT_IV_. The absorption phase rate constant (Ka) was obtained by dividing 0.693 by MAT, and the half-life of absorption (t_1/2ka_) was further determined as 0.693 × MAT. Bioavailability (F) was calculated as the ratio of AUC_PO_ to the AUC_IV_ [17].

### 2.6. Statistical Analysis

The statistical analysis of pharmacokinetic parameters was conducted using SPSS software (version: 22.0). Firstly, gender effects were examined in each treatment group. The normality of each pharmacokinetic parameter between genders within each group was assessed using the Kolmogorov–Smirnov test. Results indicated that C_max_ between PO (male) and PO (female) groups, as well as Cl and V_SS_ between IV (male) and IV (female) groups, followed a normal distribution. For these parameters, the independent sample *t*-test was utilized to analyze significance. Conversely, the Mann–Whitney U test was employed for other parameters that did not conform to a normal distribution. Gender effects were found to be nonsignificant in both treatment groups. Subsequently, the statistical analysis was performed on the pharmacokinetic parameters of both sexes within the two treatment groups. The normality between PO (male), PO (female), IV (male), and IV (female) groups was assessed using the Shapiro–Wilk test. Notably, t_1/2λz_, AUC, AUC%, AUMC, and MRT did not exhibit normal distribution. Hence, one-way ANOVA was conducted for statistical analysis, while the independent samples *t*-test was applied to the remaining parameters. Finally, a statistical analysis was conducted between the PO (all) and IV (all) groups. A significance level of *p* < 0.05 was used to determine the presence of a significant difference.

## 3. Results

The extraction and detection methods employed in this study showcased robust specificity, high accuracy and precision, as well as excellent selectivity, in detecting danofloxacin in plasma. The retention time of danofloxacin was determined to be approximately 7.2 min, effectively avoiding interference from impurity peaks. The concentration range of danofloxacin exhibited a good linear relationship and reproducibility, ranging from 0.005 to 5 µg/mL. The regression equation for the calibration curve was C = 7 × 10^−9^ S + 0.0021 (where C represents the calculated concentration of danofloxacin and S represents the peak area of danofloxacin in the chromatogram), with an R^2^ of 0.9999. Under these conditions, the recovery rate of danofloxacin ranged from 84.49% to 93.94%, the intra-day variation coefficient ranged from 1.85% to 4.31%, and the inter-day variation coefficient ranged from 2.44% to 3.56%. The LOD and LOQ were determined to be 0.0005 and 0.001 µg/mL, respectively.

**Figure 1:**
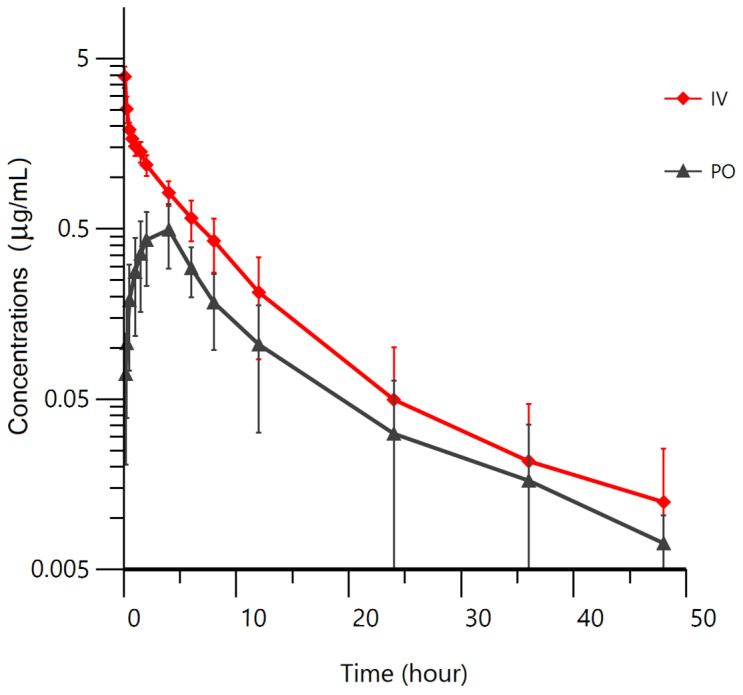
Mean ± SD plasma concentrations (μg/mL) of danofloxacin in Gushi chickens following PO (*n* = 16) and IV (*n* = 16) administration at a single dose of 5 mg/kg BW.

Throughout the entire experimental period, the 32 Gushi chickens remained in good condition with no observed adverse reactions after administration. At the last plasma collection point (48 h), danofloxacin was still detectable and exceeded the LOQ. The plasma concentration of danofloxacin in Gushi chickens after PO and IV administration was graphically represented in Figure 1, while the main pharmacokinetic parameters obtained through NCA were displayed in Table 1. The AUC_0-∞_ after PO and IV administration was determined to be 4.72 ± 1.82 and 11.76 ± 3.25 h·µg/mL, respectively, exhibiting a significant difference. The MRT was calculated as 10.20 ± 2.47 h (PO) and 7.05 ± 1.97 h (IV), with a significant difference. The elimination half-life after PO and IV administration was 11.24 ± 3.90 and 10.17 ± 3.72 h, respectively, showing no significant difference. Following IV administration, danofloxacin demonstrated a wide distribution and slow elimination in Gushi chickens, with Vz of 6590.63 ± 2900.10 mL/kg and Cl of 446.86 ± 89.05 mL/h/kg. After PO administration, the drug concentration reached a peak of 0.53 ± 0.19 µg/mL at 4 h. The MAT was determined as 3.15 ± 2.47 h, but the bioavailability (F) was found to be only 40.12 ± 15.83%.

As shown in Table 1, we conducted a separate analysis of the pharmacokinetic characteristics of danofloxacin in male and female Gushi chickens for each route of administration. However, we observed no gender effect on any of the pharmacokinetic parameters under both routes.

## 4. Discussion

This study is the first to examine the pharmacokinetics of danofloxacin in Gushi chickens, and danofloxacin was given at 5 mg/kg via PO and IV routes. In a pharmacokinetic study, important parameters for assessing drug absorption rate include C_max_, T_max_, and t_1/2ka_. In the current study, danofloxacin reached a peak concentration of 0.53 ± 0.19 µg/mL at 4 h after PO administration, with a t_1/2ka_ value of 2.37 ± 1.60 h. Under the same dosage (5 mg/kg) in different poultry species, the current C_max_ was lower than that in Jing Hong hens (1.05 µg/mL) [12], Muscovy ducks (0.81 µg/mL) [18], and Bilgorajska geese (0.96 µg/mL) [11]. However, the peak concentration was similar to that found in Lingnan yellow broiler chickens (0.51 µg/mL) [19], Lohmann broiler chickens (0.47 µg/mL) [20], and Arbor Acres broilers (0.53 µg/mL) [21], following the same dose administration. It is worth noting that the Gushi chickens had a later T_max_ compared to all the aforementioned poultry varieties (1.73, 1.21, 1.7, 2.33, 1.5, and 2.92 h, respectively). Gushi chickens are usually raised in a free-range environment, which allows them to roam freely. This exercise habit may have caused its T_max_ to be delayed more than the other birds. In this study, Gushi chickens were initially raised in a free-range environment before being placed in cages for the experiment. They underwent a 14-day acclimatization period in the new environment.

The elimination of danofloxacin in Gushi chickens occurred at a slow pace. The half-life (t_1/2λz_) after PO and IV administration was 11.24 ± 3.90 and 10.17 ± 3.72 h, respectively, with no significant difference. While the current PO and IV t_1/2λz_ values in Gushi chickens were longer than those in Lohmann broiler chickens (6.62 and 6.73 h, respectively) [20] and turkeys (9.74 and 8.64 h, respectively) [22]. However, in all previous studies, there were no significant differences between the IV and extravascular t_1/2λz_ values. This phenomenon is not only observed in poultry but also in livestock. For example, in donkeys, the t_1/2λz_ after IV and intramuscular (IM) administration was 7.25 and 6.36 h, respectively [23], without significant differences. In sheep, the t_1/2λz_ after IV and subcutaneous (SC) administration was 3.27 and 3.07 h, respectively [24], with no statistical difference. These findings suggest that the routes of administration have a limited impact on the in vivo elimination of danofloxacin in the same animal.

After IV administration, Gushi chickens exhibited a volume of distribution at steady state (V_SS_) of 3035.00 ± 428.84 mL/kg, which was lower than those observed in Lohmann broiler chickens (10,200 mL/kg) [20], Japanese quails (8570 mL/kg) [10], and Jing Hong hens (3500 mL/kg) [12]. On the other hand, other fluoroquinolones, such as marbofloxacin (1247 mL/kg) in Japanese quail [25] and enrofloxacin (1060 mL/kg) in cattle [26], demonstrated lower V_SS_ values. The wider distribution of danofloxacin compared to other fluoroquinolones may be attributed to its higher lipid solubility and intracellular permeability [27]. Additionally, as free-range breeds, Gushi chickens have lower fat content compared to cage breeds, which may contribute to the decreased distribution of danofloxacin in their bodies compared to other breeds.

After PO administration, the MRT value was 10.20 ± 2.47 h, longer than that of IV administration (7.05 ± 1.97 h), and the difference between both was calculated to be the MAT (3.15 ± 2.47 h). The PO t_1/2ka_ was further calculated as 2.37 ± 1.60 h, which was longer than those in Japanese quails (0.98 h) [10] and Muscovy ducks (0.67 h) [18], but shorter than those in guinea fowls (3.45 h) [10], Chukar partridge (3.60 h) [4] and common pheasants (4.90 h) [10].

After administering 5 mg/kg of danofloxacin through IV administration, the AUC_0-∞_ in Gushi chickens was found to be 11.76 h·µg/mL. This is comparable to the AUC_0-∞_ observed in Jing Hong hens (10.41 h·µg/mL) [12] and turkeys (11.76 h·µg/mL) [22]. However, it is lower than that reported in Bilgorajska geese (15.35 h·µg/mL) [11], but higher than those in Muscovy ducks (5.53 h·µg/mL) [18] and Lohmann broiler chickens (3.55 h·µg/mL) [20] which recorded lower values. On the other hand, when danofloxacin was administered orally, the AUC_0-∞_ was only 4.72 h·µg/mL. The current result is consistent with the values observed in most birds (5.7, 4.89, 3.53, 5.2 h·µg/mL) [18,21,28,29]. The bioavailability (F) of danofloxacin was found to be only 40.12% in the present study. This value is lower when compared to Chukar partridges (47.62%) [4], swan geese (57.68%) [30], Bilgorajska geese (57.95%) [11], Muscovy ducks (89.26%) [18], Lohmann broiler chickens (99.2%) [21], and Jing Hong hens (101.26%) [12]. The low bioavailability observed in Gushi chickens can be attributed to lifestyle and population differences.

Upon comparing all the pharmacokinetic data, we observed that the gender of Gushi chickens had minimal impact on the pharmacokinetics of danofloxacin. Despite the larger body weight of males in comparison to females (2.14 versus 1.60 kg; average body weights), the pharmacokinetic parameters displayed only slight variations between the two sexes. However, these differences were not statistically significant when comparing males and females under the same administration route. It is worth mentioning that previous studies exploring the pharmacokinetics of danofloxacin in other birds did not separate the data based on gender [7,22]. This could be attributed to the findings of similar results in other avian species.

In our experiments, we overlooked the possible effects of danofloxacin on chicken blood parameters. However, according to a report by FAO, female dogs that were orally administered danofloxacin at a dose of 50 mg/kg BW per day for two weeks showed increased levels of serum blood urea nitrogen and creatinine [31]. This suggests that danofloxacin may have an impact on specific blood parameters in dogs. However, it is important to highlight that directly applying these findings to chickens may not be appropriate. To fully comprehend the potential effects of danofloxacin on Gushi chickens and their blood parameters, further research specifically tailored to this species is required.

A previous study conducted on turkeys found that an AUC/MIC ratio greater than 6.73 h could effectively eradicate *Escherichia coli* [22]. In addition, the previous investigation noted that danofloxacin demonstrated an MIC50 of 0.25 µg/mL against poultry *Escherichia coli* [32] and an MIC of 0.05 µg/mL against *Pasteurella multocida* [19]. Assuming that the MIC values for *Escherichia coli* and *Pasteurella multocida* in Gushi chickens are consistent with the findings of previous studies, the current oral dosage of 5 mg/kg would yield an AUC/MIC ratio of 18.88 h and 94.4 h against *Escherichia coli* and *Pasteurella multocida*, respectively—both ratios surpassing 6.73 h. Since the same IV dose resulted in a significantly higher AUC (Table 1), the therapeutic threshold of 6.73 h was more easily reached with the current IV dose. These results suggest that both PO and IV administration of danofloxacin at 5 mg/kg to Gushi chickens may be effective treatments for infections caused by *Escherichia coli* or *Pasteurella multocida*.

## 5. Conclusions

The current study demonstrated excellent pharmacokinetic profiles for danofloxacin, including quick absorption, wide distribution, and slow elimination in Gushi chickens. However, incomplete oral absorption was also observed in Gushi chickens with absolute bioavailability at 40.12 ± 15.83%. Based on the ratio of AUC/MIC determined in the current study, an intravenous or oral dose of 5 mg/kg danofloxacin would be expected to successfully treat Gushi chickens infected by *Escherichia coli* or *Pasteurella multocida*.

## Figures and Tables

**Table 1:** Pharmacokinetics parameters of danofloxacin in Gushi chickens after oral (PO; *n* = 8 males and 8 females), and intravenous (IV; *n* = 8 males and 8 females) administration at a single dose of 5 mg/kg BW.

Parameters	Unit	PO (All)	PO (Male)	PO (Female)	IV (All)	IV (Male)	IV (Female)
λz	1/h	0.07 ± 0.02 ^A^	0.06 ± 0.02 ^a^	0.07 ± 0.02 ^a^	0.08 ± 0.03 ^A^	0.08 ± 0.03 ^a^	0.08 ± 0.03 ^a^
t_1/2λz_	h	11.24 ± 3.90 ^A^	12.30 ± 3.35 ^a^	10.17 ± 4.33 ^a^	10.17 ± 3.72 ^A^	10.12 ± 3.92 ^a^	10.23 ± 3.78 ^a^
T_max_	h	4	4 ^a^	4 ^a^	NA	NA	NA
C_max_	µg/mL	0.53 ± 0.19	0.50 ± 0.19 ^a^	0.57 ± 0.20 ^a^	NA	NA	NA
C_0_	µg/mL	NA	NA	NA	4.67 ± 1.15	5.03 ± 0.74 ^a^	4.32 ± 1.42 ^a^
AUC_0-∞_	h·µg/mL	4.72 ± 1.86 ^A^	3.82 ± 1.33 ^a^	5.62 ± 2.07 ^a^	11.76 ± 3.25 ^B^	11.02 ± 2.48 ^b^	12.50 ± 3.90 ^b^
AUC%	%	2.34 ± 1.28 ^A^	2.60 ± 1.32 ^a^	2.08 ± 1.27 ^a^	1.29 ± 1.18 ^B^	1.20 ± 0.88 ^a^	1.39 ± 1.48 ^a^
AUMC_0-∞_	h^2^·µg/mL	49.94 ± 29.66 ^A^	37.14 ± 12.67 ^a^	62.74 ± 36.74 ^ab^	88.03 ± 60.99 ^B^	75.94 ± 21.52 ^b^	100.11 ± 84.69 ^b^
MRT	h	10.20 ± 2.47 ^A^	9.81 ± 1.91 ^ab^	10.58 ± 3.02 ^a^	7.05 ± 1.97 ^B^	6.83 ± 0.66 ^bc^	7.26 ± 2.78 ^c^
Cl	mL/h/kg	NA	NA	NA	446.86 ± 89.05	470.00 ± 91.03 ^a^	423.75 ± 86.51 ^a^
V_Z_	mL/kg	NA	NA	NA	6590.63 ± 2900.10	7056.25 ± 3354.05 ^a^	6125.00 ± 2505.47 ^a^
V_SS_	mL/kg	NA	NA	NA	3035.00 ± 428.84	3193.57 ± 528.39 ^a^	2876.25 ± 239.34 ^a^
MAT	h	3.15 ± 2.47	2.15 ± 1.25 ^a^	4.18 ± 2.64 ^a^	NA	NA	NA
Ka	h	0.32 ± 0.26	0.39 ± 0.32 ^a^	0.24 ± 0.14 ^a^	NA	NA	NA
t_1/2ka_	h	2.37 ± 1.60	1.92 ± 1.32 ^a^	2.88 ± 1.83 ^a^	NA	NA	NA
F	%	40.12 ± 15.83.	32.45 ± 9.60 ^a^	47.78 ± 17.62 ^a^	NA	NA	NA

Note: T_max_ results are presented as medians. Results for other parameters are expressed as Mean ± SD. Within a row, values not sharing a common superscript letter are significantly different (uppercase versus uppercase, lowercase versus lowercase; *p* < 0.05). λz, the first-order rate constant associated with the terminal phase; AUC_0–∞_, the area under the concentration–time curve from the time of dosing to infinity; AUC%, the percentage from the last data point to the concentration–time curve infinity; AUMC_0–∞_, the area under the moment curve from the time of dosing to infinity; C_0_, initial concentration; Cl, total body clearance; C_max_, observed peak concentration; F, absolute bioavailability after oral administration; Ka, the absorption phase rate constant; MAT, mean absorption time; MRT, mean residence time extrapolated to infinity; t_1/2λz_: apparent elimination half-life; t_1/2ka_: the half-life of absorption; T_max_: time to reach peak concentration; V_Z_, the volume of distribution; V_SS_, the volume of distribution at steady state; NA, not applicable.

## Data Availability

The data that support the study findings are available upon request and after authorization by the authors. The data are not publicly available due to privacy.
